# Surface-Mediated Molecular Transport of a Lipophilic Fluorescent Probe in Polydisperse Oil-in-Water Emulsions

**DOI:** 10.1021/acs.langmuir.2c02597

**Published:** 2023-03-15

**Authors:** Marius R. Bittermann, Tatiana I. Morozova, Santiago F. Velandia, Elham Mirzahossein, Antoine Deblais, Sander Woutersen, Daniel Bonn

**Affiliations:** † Van der Waals-Zeeman Institute, IoP, University of Amsterdam, Science Park 904, 1098 XH Amsterdam, Netherlands; ‡ Institut Laue-Langevin, 71 Avenue des Martyrs, Grenoble 38042, France; § Van ’t Hoff Institute for Molecular Sciences, University of Amsterdam, Science Park 904, 1098 XH Amsterdam, Netherlands

## Abstract

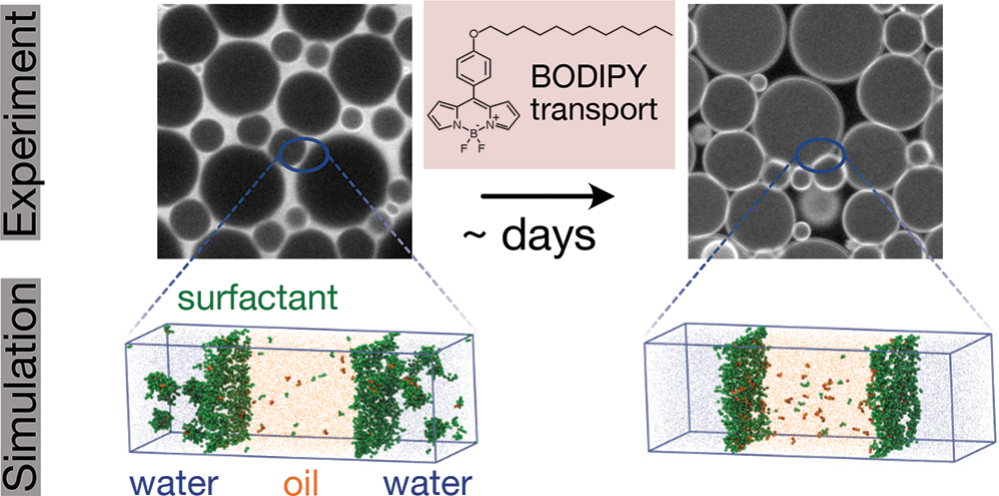

Emulsions often act as carriers for water-insoluble solutes that are delivered to a specific target. The molecular transport of solutes in emulsions can be facilitated by surfactants and is often limited by diffusion through the continuous phase. We here investigate this transport on a molecular scale by using a lipophilic molecular rotor as a proxy for solutes. Using fluorescence lifetime microscopy we track the transport of these molecules from the continuous phase toward the dispersed phase in polydisperse oil-in-water emulsions. We show that this transport comprises two time scales, which vary significantly with droplet size and surfactant concentration, and, depending on the type of surfactant used, can be limited either by transport across the oil–water interface or by diffusion through the continuous phase. By studying the time-resolved fluorescence of the fluorophore, accompanied by molecular dynamics simulations, we demonstrate how the rate of transport observed on a macroscopic scale can be explained in terms of the local environment that the probe molecules are exposed to.

## Introduction

In its simplest form, an emulsion is a surfactant-stabilized mixture of immiscible liquids, in which one phase is dispersed in the other.^1^ One increasingly popular application of emulsions is as delivery systems for bioactive solutes, such as for drugs^2−4^ or for functional food ingredients.^5^ Considering drug delivery, one has to keep in mind that most newly discovered drugs are lipophilic, i.e., poorly soluble in water.^6−8^ For such drugs, emulsions tend to be promising delivery agents, given that their oil phase solubilizes lipophilic drugs while they retain a high bioavailability.^4^ As an example, in a recent study oil-in-water emulsions were shown to be promising delivering agents for the topical delivery of the lipophilic drug bifonazole.^9^ From a thermodynamic viewpoint, emulsions are complex; they are in a metastable state stabilized by surfactants, molecules that adsorb to the interface between the oil and aqueous phases. When aging, emulsions tend to destabilize, which involves mechanisms such as flocculation, creaming, or coalescence. Emulsion aging is also accompanied by a material flow that can be composed of the dispersed phase itself, in a process termed Ostwald ripening,^10,11^ or of solutes being exchanged between the phases.^12−17^ For emulsions that act as delivery systems for solutes, a good understanding of the dynamics of such molecular transports is crucial, given that the solutes partition between the phases of the emulsions and eventually have to be delivered to a target. Baret and co-workers have shown that for monodisperse water-in-oil emulsions the exchange of solutes, poorly soluble in the continuous phase, is mediated by micelles and limited by diffusion through the continuous phase.^16^ The diffusive process was demonstrated to be faster with increasing surfactant concentration but slower with increasing spacing between the droplets.

Here, we investigate the transport of a lipophilic molecule solubilized in micelles in the continuous phase to the interior of the droplets in a polydisperse oil-in-water emulsion. As a model system for lipophilic solutes, we use the dye molecule BODIPY-C12, a popular probe to study membranes on the nanoscale.^18−20^ By tracking BODIPY-C12 in these emulsions, we find that the molecular transport from the continuous phase into the oil droplets is a two-step process: the slow depletion of BODIPY-C12 from the continuous phase is followed by dye exchange between the oil droplets. The rate of transport shows a dependence on surfactant concentration and droplet size, which we explain using simple models based on permeability theory and diffusion. We find that for some surfactants the partitioning of the solute molecules at the surface of droplets can become the limiting step, where the solute exchange is slowed to a time scale of days. Surprisingly, in our system the dynamics of the transport can be explained by neither the surfactant polarity (i.e., the hydrophilic–lipophilic balance) nor electrostatic interactions. Instead, analysis of the time-resolved fluorescence of the fluorophore suggests that the retention at the oil–water interface is due to interactions on a molecular level, in particular due to the mobility of BODIPY-C12 in its local environment and the size of the surfactant molecules. To push further our understanding of the process, we perform coarse-grained molecular dynamics simulations that confirm the experimental observations. Additionally, simulation results suggest that the parameters governing this surface-mediated molecular transport are the interactions between the head groups of the dye and surfactant molecules.

## Methods

### Emulsion Preparation

**Figure 1:**
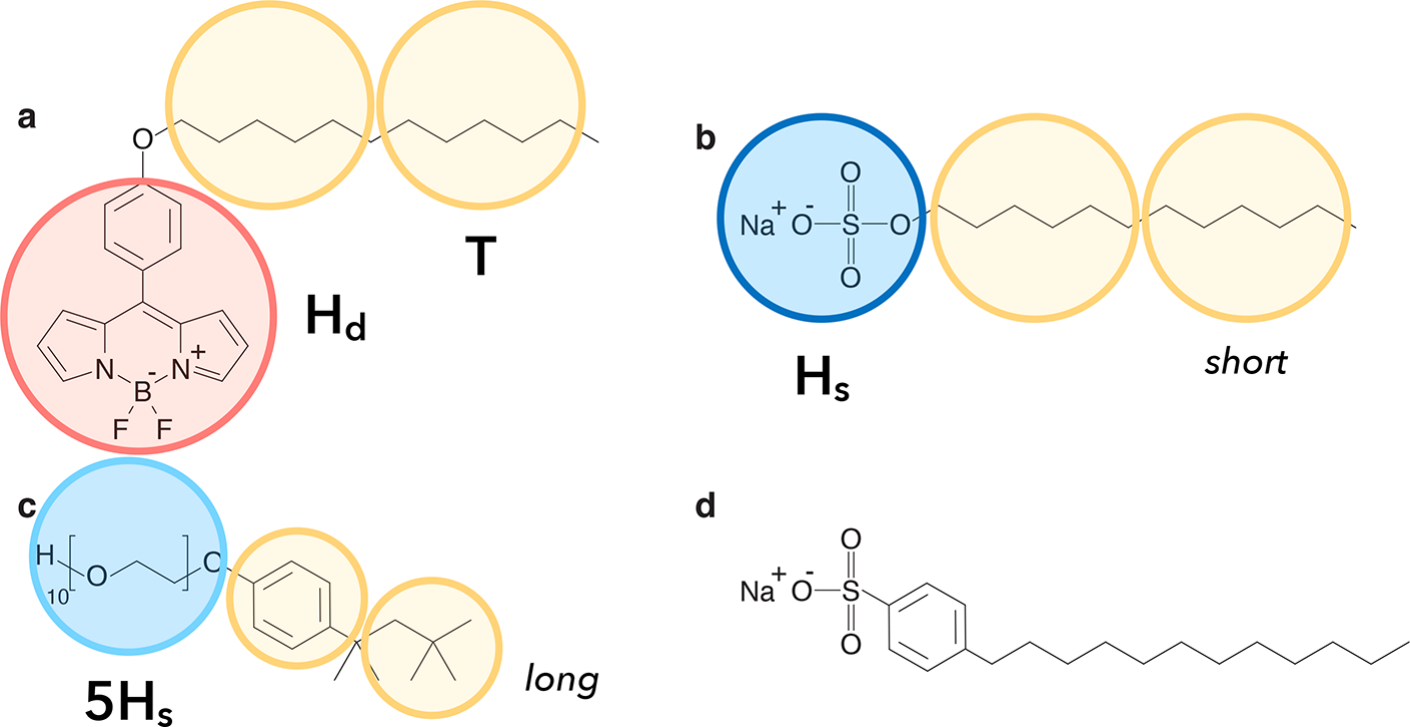
Chemical structures of the molecular rotor BODIPY-C12 (a) and the surfactants SDS (b), TX-100 (c), and SDBS (d). Schematic representations of the corresponding simulation models and the bead types are shown as well. Note that all beads have the same size in simulations.

Emulsions were prepared by dispersing viscous polydimethylsiloxane silicone oil (500 cst, from Sigma-Aldrich) in aqueous sodium dodecyl sulfate (SDS, ≥99.0%, from Sigma), sodium dodecylbenzenesulfonate (SDBS, technical grade, from Sigma-Aldrich), and *t*-octylphenoxypolyethoxyethanol (TX-100, laboratory grade, from Sigma-Aldrich) solutions. We chose an oil volume fraction of 80% and surfactant concentrations of 1 and 2 wt %, which are well above the critical micelle concentrations (≈4 and ≈8 × cmc, respectively).^21^ Using a Silverson high-shear industrial mixer at 6000 rpm for ≈20 min, we produced polydisperse oil-in-water emulsions,^22,23^ which remained stable for the duration of the experiments (Figure S1) and beyond. The fluorophore BODIPY-C12 was synthesized using the method by Lindsey and Wagner.^24^ Prior to the preparation of the emulsions, a stock solution of BODIPY-C12 in ethanol was diluted with the continuous phase (1:100) to obtain a dye concentration of ≈1 μM. We anticipate the presence of ethanol not to impact the experiments considering its strong dilution. BODIPY-C12 dissolves in micellar solution, and in oil, but is poorly soluble in water (Figure S2). The chemical structures of the dye molecule and surfactants are drawn in Figure 1.

### Fluorescence Lifetime Imaging Microscopy

All fluorescence lifetime imaging microscopy (FLIM) measurements were performed using a Leica TCS SP8 HyD confocal fluorescence lifetime microscope. As an excitation source we used a 470 nm pulsed laser at 40 MHz, and the emitted light was detected in the range of 500–700 nm by a Hyd detector. For all experiments we used a 100× magnification in-oil objective with a numerical aperture of 1.25. Images were acquired at a scan speed of 100 Hz and accumulated 4 times each, in the course of several hours after sample preparation, and for up to six consecutive days after. From these images we extracted both the fluorescence intensity, which is proportional to the concentration of the dye *c* (Figure S3), and the fluorescence lifetime τ, which provides information on the local environments the dye molecules are exposed to.^18−20,25,26^ For the analysis of the fluorescence intensity of the microscopy images we used the image processing package Fiji^27^ along with the collection of plugins MorpholibJ^28^ and the ellipse splitting plugin.^29^ We refer to the Supporting Information for a more detailed description of the image analysis. The fluorescence lifetime was analyzed using the Leica Application Suite X. For each analyzed phase we included at least 10^4^ photon counts. The time-resolved fluorescence was fitted using a model based on *n*-exponential reconvolution. For the fit of multiexponential decays we used the amplitude-weighted average lifetime, . We considered fits acceptable for χ^2^ < 1.5.

### Simulation Model and Methods

To reach the length and time scales associated with the diffusion of the dye molecules through the oil–water interface and capture experimental trends in a qualitative manner, we chose to model the systems using a coarse-grained (CG) description. These calculations using the atomistic resolution of the compounds would be computationally unfeasible. Additionally, to the best of our knowledge, there is no full atomistic model for BODIPY-C12 and TX-100 molecules that are compatible with CG models of water, SDS, and silicone oil. Thus, we performed CG molecular dynamics simulations of systems containing a solution of surfactant and dye molecules in a mixture of water and oil.

As we aimed for a generic model, we deliberately did not take into consideration the shape anisotropy of the solvent particles and modeled them explicitly as spherical beads of unit diameter σ and unit mass *m*. The interaction between solvent particles was modeled via the Lennard-Jones (LJ) potential
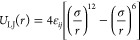
1where *r* is the distance between a pair of particles and ε_*ij*_ controls the interaction strength between particles of type *i* and *j*. For particle pairs of the same type, we used ε_oo_ = ε_ww_ ≡ ε = *k*_B_*T*, where *k*_B_ is the Boltzmann constant and *T* is the absolute temperature. The cutoff radius of the LJ potential was set to *r*_cut_ = 3.0σ. The interspecies interaction was modeled using the purely repulsive Weeks–Chandler–Andersen (WCA) potential, achieved by truncating *U*_LJ_(*r*) at its minimum *r*_min_ = 2^1/6^σ and shifting it by ε.^30^ At the simulation conditions employed, the two fluids are immiscible, which leads to the formation of a liquid–liquid interface.^31^ This strategy was shown to be robust for modeling the immiscibility of two fluids,^31,32^ including oil–water interfaces.^33^

We approximated both dye and surfactant molecules as chains made up of several beads connected through springs, which were modeled via the finitely extensible nonlinear elastic (FENE) potential combined with the WCA potential. We used the standard Kremer–Grest parameters for the FENE potential to prevent unphysical bond crossing.^34^ In a recent numerical study on the oil–water interfaces decorated by surfactant molecules, a CG mapping for water and SDS molecules was proposed.^35^ In this representation, five water molecules were lumped together into one bead. Thus, the volume occupied by five water molecules at room temperature, *v*_w_ = 0.15 nm^3^, defines the volume of one bead in the simulations. The hydrophobic tail of SDS is equivalent to two water beads in size and is represented by two beads (bead type T). The headgroup (bead type H_s_) was modeled by a single bead resulting in three beads per surfactant (*n*_s_ = 3). Because SDS and BODIPY-C12 molecules are quite similar with respect to their chemical structure (compare Figures 1a and 1b), i.e., both molecules are composed of a hydrophobic tail containing 12 carbon atoms and a headgroup (bead type H_d_), we used the same mapping for both BODIPY-C12 and SDS. To investigate how the length of the headgroup of a surfactant molecule influences the diffusion of dye molecules, we additionally introduced a surfactant type that is similar to TX-100 (Figure 1c). The hydrophobic tail of TX-100 was also modeled by two T-type beads, as its length is similar to the hydrophobic tails of SDS or BODIPY-C12 molecules. The headgroup of TX-100, however, is a poly(ethylene oxide) (PEO) chain. For ten monomers, the Kuhn length for PEO is ≈0.68 nm, which roughly equals the size of two monomers.^36^ Because in our simulations the unit of length , we modeled the headgroup of TX-100 using five H_s_ beads (*n*_s_ = 7). A schematic mapping and the detailed summary of bead types are provided in Figure 1.

Nonbonded interactions between all bead types were also modeled using the *U*_LJ_ potential introduced above. As the parameter choice is crucial to adequately model the system, we chose the strength of the potential ε_*ij*_/ε based on the available experimental information to capture the relative strength between the compounds. The resulting values are summarized in Table 1.

**Table 1:** Values of the Interaction Strength ε_*ij*_/ε Used in the Pair Potential *U*_LJ_ to Model Nonbonded Interactions between *i* and *j* Particle Types

	W	O	H_s_^short^/H_s_^long^	H_d_	T
W	1.0	WCA	1.9/1.1	0.9	0.2
O		1.0	0.2	0.9	0.9
H_s_^short^/H_s_^long^			1.0	3.6/0.225	0.2
H_d_				1.0	0.9
T					1.0

For the intraspecies interaction, we used . In our simplified modeling approach, we use a single interaction strength, ε_min_ = 0.2ε, to capture the immiscibility between some species in the system: H_s_/O, H_s_/T, and T/W. Because the headgroup of the dye molecule is miscible in both types of solvents, we set up the interactions between the headgroup and solvents as .^37^ For the oil-soluble tail of both the dye and surfactant molecule, we also chose  to be equal to 0.9. Experimentally, the surfactant molecules are found either at the oil–water interface screening the interaction between the immiscible fluids or in aqueous solution. To achieve such a scenario in the simulations, we set the interaction strength  between the water beads and the headgroup of the surfactants H_s_ to 1.9ε and 1.1ε for the three- and seven-bead surfactant models, respectively. This choice of interaction parameters results in a similar interfacial coverage by the two surfactant types (Figure S4).

To qualitatively capture the hydrophilic strength of the surfactant headgroups employed in the experiments, we make use of their HLB values.^38^ As we represent the SDS headgroup by a single bead (short), while using five beads for the headgroup of TX-100 (long) (Figure 1b,c), the ratio of the interaction strength is then estimated as . Thus, we choose  to be equal to 3.6 and 0.225 for short and long surfactant chains, respectively.

To model the interface, we chose a simulation box elongated along the *z*-direction. The box size was set to *L*_*x*_ = *L*_*y*_ = 36σ and *L*_*z*_ = 3*L*_*x*_ = 108σ. Because of the periodic boundary conditions, there are two interfaces within the simulation box. In our simulations we determined the position of the interface between the water and oil phases from the maximum of the surfactant concentration profile along the *z*-axis.^39^ The overall particle number density was set to ρ = 0.66σ^–3^.^33^ We chose the composition of the solvent mixture as 50:50, leading to approximately 43000 particles for each type of liquid. As in the experiments, we assumed that the liquid–liquid interface is saturated with surfactant molecules. We estimate the number of surfactant required as *N*_s_ = 2*L*_*x*_*L*_*y*_*l*_u_^2^/*A*_s_, where *A*_s_ ≈ 0.5 nm^2^/molecule is the experimentally determined surface area per SDS molecule adsorbed at the interface between silicone oil and water.^40^ The resulting values for *N*_s_ is 2253 surfactant molecules per simulation for the surfactant model based on three beads. This leads to the surfactant concentration *c*_s_ = *n*_s_*N*_s_/*V*_box_ = 0.05σ^–3^, which is close to the experimental values. For the seven-bead surfactant model, we kept the same concentration resulting in *N*_s_ = 966 chains per simulation. We then added *N*_d_ = 100 dye molecules into the system to investigate their diffusive behavior. In total, the systems are composed of 92378 beads.

Starting configurations were generated by randomly placing all oil beads in one-half of a simulation box, while placing the remaining beads in the second half. We followed a multistep equilibration procedure. First, we ran a short simulation for 1 × 10^6^ time steps where only the position of the liquid beads was integrated to equilibrate the phase-separated fluids. Next, we achieved a homogeneous distribution of surfactant molecules in a box by simulating a system for 1 × 10^7^ time steps in which the interaction strength between the T, H_s_ beads, and liquids was set to ε. Afterward, a simulation of 3.5 × 10^7^ time steps was conducted in which the surfactant and solvent beads interact with the parameters presented in Table 1. This simulation results in the formation of a liquid–liquid interface decorated by surfactants. To keep the dye molecules in the aqueous phase during the equilibration stage, we set a purely repulsive interaction (WCA) between the H_d_ and T beads and the oil phase. Finally, we set all interactions according to Table 1 and conducted a production run of 2 × 10^8^ time steps. Three independent runs were performed for each value of the interaction strength . Simulations were conducted in the *NVT* ensemble at *T* = 1 using a Nosé–Hoover thermostat. The equations of motion were integrated using the velocity-Verlet algorithm with a time step of Δ*t* = 0.005τ, where  is the intrinsic MD unit of time. All simulations were performed using the HOOMD-blue simulation package (v.2.9.2).^41^

## Results and Discussion

### Experimental Results

**Figure 2:**
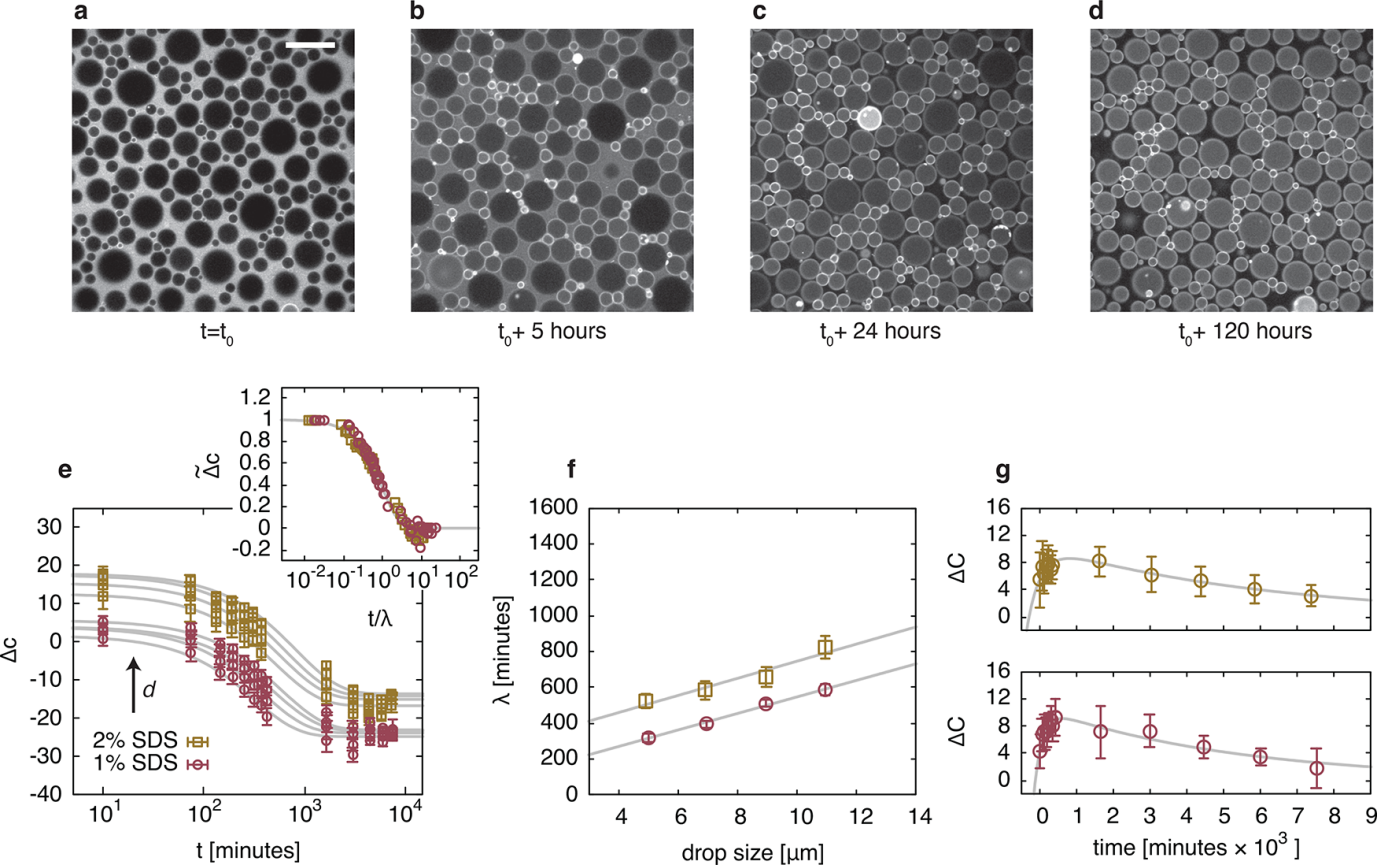
Molecular transport in an emulsion. (a–d) Fluorescence microscopy images show the molecular transport of the lipophilic dye BODIPY-C12 from the continuous phase toward the dispersed phase of a model oil-in-water emulsion stabilized with 1% SDS. Once the continuous phase is depleted of BODIPY-C12 (after ≈24 h), the fluorophore is also transported between oil droplets. The whole exchange takes several days. The scale bar is 20 μm. (e) We quantify this exchange using the parameter Δ*c*, which gives the difference in concentration of dye molecules in the continuous phase and in the oil droplets: *c*_w_ – *c*_0_. For different oil droplet sizes, *d* (average drop sizes: ⟨*d*⟩ ≈ 5, 7, 9, and 11 μm), Δ*c* decays exponentially and is fitted using eq 2. Normalizing Δ*c* by its initial and final value, and rescaling this normalized concentration difference using a single time scale λ, collapses all data (inset). (f) The time scale λ increases linearly with droplet size with a slope independent of surfactant concentration. (g) To quantify the exchange between the oil droplets, we use the parameter Δ*C*, which gives the difference in concentration of dye molecules in oil droplets of different sizes: Δ*c*(⟨*d*⟩ = 5 μm) – Δ*c*(⟨*d*⟩ = 11 μm). The dynamics of Δ*C* as a function of time reveal two time scales for both emulsions stabilized with 2% (top) and 1% SDS (bottom). Initially, Δ*C* grows as the smaller droplets fill up faster (inset). Then, as the continuous phase is depleted of dye molecules, BODIPY-C12 is being transported from concentrated small droplets toward less concentrated large droplets. This process happens at a time scale of days. The fit is the exponential model presented in eq 3.

In the case of SDS stabilized emulsions, at *t*_0_, which for all samples is ≈10 min after emulsion preparation, we detect the fluorophore in the continuous phase only (Figure 2a), where it is loaded onto micelles (Figures S2 and S5). BODIPY-C12 then diffuses from the initially swollen micelles into the oil droplets, which happens gradually over the course of 24 h (Figure 2b,c), with a rate that is dependent on the size of the oil droplets; the smaller droplets “fill up” faster. In addition, we observe highly fluorescent regions at the periphery of the droplets, which suggests aggregation of the dye molecules at the oil–water interface.^42−44^ After the continuous phase is depleted of BODIPY-C12 (Figure S6), we find the dye diffusing from the brighter small droplets toward the empty larger ones until the fluorescence intensity is uniform among the oil phase (Figure 2d). This molecular transport is not limited to an emulsified system and also occurs in bulk (Figure S7).

We characterize the exchange of dye by first analyzing the temporal evolution of the concentration difference Δ*c* = *c*_w_ – *c*_0_, which is defined as the difference between the concentration of dye molecules in the water phase, *c*_w_, and the concentration of dye molecules dissolved in the silicone oil droplets, *c*_0_. To obtain the concentration from the fluorescence intensity, we need to know the different extinction coefficients of BODIPY-C12 in micellar solution and in oil. We therefore first measure the fluorescence intensity of the dye in both neat phases separately and then correct the concentrations accordingly.

To analyze the dependence of the exchange on the oil droplet size, *d*, we binned Δ*c*(*d*) into four intervals (Figure S8) to obtain Δ*c*(*t*) for the average droplet sizes ⟨*d*⟩ ≈ 5, 7, 9, and 11 μm. A plot of Δ*c* versus time (Figure 2e) reveals exponential decays that we fit using
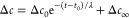
2Rescaling the normalized data  between Δ*c*(*t*_0_) = Δ*c*_0_ and Δ*c*(*t*_*∞*_) = Δ*c*_∞_ (measured at day 6) by the time scale λ produces a master curve (Figure 2e, inset). This exponential relaxation is in agreement with theory based on diffusive transport facilitated by surfactants; the transport is governed by thermodynamics and dictated by differences within the chemical potential of the solute over which the system equilibrates.^15,16^ The time scale of the transport was previously found to be determined by the permeability *P* of the micellar phase, the droplet volume *V*, and surface area *S* and can be expressed as λ = *V*/(*SP*).^15,16^ This relation predicts the time scale to scale linearly with droplet size, which our data confirm (Figure 2f), albeit with an offset λ_0_ that varies with the surfactant concentration. Using the slope of λ(⟨*d*⟩) (fit in Figure 2f), we derive a permeability *P*, defined as the diffusion rate of the dye molecules through the micellar phase, which, regardless of surfactant concentration, is on the order of 10^–10^ m s^–1^.

We first investigate whether the transport is limited by diffusion of the dye through the continuous phase. In this case, the permeability can be expressed as ,^45^ with  the partition coefficient, *D*_m_ the diffusion coefficient of the micelle–dye aggregates, and *l* the thickness of the interface. From the fluorescence images at equilibrium we estimate *K* ≈ 4 and *K* ≈ 2 for the emulsions stabilized with 2% SDS and 1% SDS, respectively. In addition, we find that the ratio in λ_0_ between 2% and 1% SDS is given by the ratio between surfactant concentration in the continuous phase, which has been found to scale linearly with the partition coefficient *K*.^16^ Micelles formed by SDS were shown to be ≈10^–9^m in diameter^46^ and are thus, according to the Stokes–Einstein equation, expected to diffuse at *D*_m_ ≈ 10^–10^ m^2^ s^–1^. Then, estimating the diffusing object to cross an interface of nanometric thickness *l*, we predict permeabilities a factor of 10^9^ larger than what we experimentally observe.

We thus conclude that for the molecular transport of BODIPY-C12 in our oil-in-water emulsions stabilized by SDS, crossing the oil–water interface is the rate-limiting step, not micellar diffusion through the continuous phase. Once the continuous phase is depleted of fluorophore, we observe exchange of the dye between the droplets (compare Figures 2c and 2d).^15,16^ To quantify this, we define the concentration difference between the largest and smallest droplets within the samples, i.e., Δ*C* = Δ*c*(⟨*d*⟩ ≈ 5 μm) – Δ*c*(⟨*d*⟩ ≈ 11 μm). Irrespective of surfactant concentration, Δ*C* first increases as a consequence of the depleting continuous phase (Figure 2g), in agreement with the data shown in Figure 2e. Subsequently, Δ*C* goes through a maximum Δ*C*_max_ followed by relaxation to Δ*C* → 0. Using the previous arguments based on diffusion, we model this using
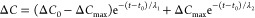
3which combines the depletion of the continuous phase with the exchange of the dye molecules between the droplets. The initial uptake occurs on a time scale of λ_1_ ≈ 400 and 200 min for the emulsions stabilized with 2% and 1% SDS, respectively. These values are as expected, similar to the ones measured for the relaxation of Δ*c* between the continuous phase and the oil droplets (Figure 2f), i.e., λ obtained from Δ*c* ≈ λ_1_ obtained from Δ*C*. The relaxation describing the exchange between droplets happens on much longer time scales of λ_2_ ≈ 6200 min for 2% SDS and 5400 min for 1% SDS. We note that the nonoverlapping values for Δ*c*(*t*_*∞*_) and the fact that Δ*C* > 0 at *t*_∞_ suggest that the smaller droplets still exhibit slightly higher concentrations in dye. This small, but noticeable effect might arise from either experimental errors or the possibility that the system did not fully reach equilibrium at the last measuring point. Considering the structural similarities of BODIPY-C12 and SDS with respect to their nonpolar alkyl moieties (compare Figures 1a and 1b), it is reasonable to assume that dye–surfactant interactions affect the transport rate.

**Figure 3:**
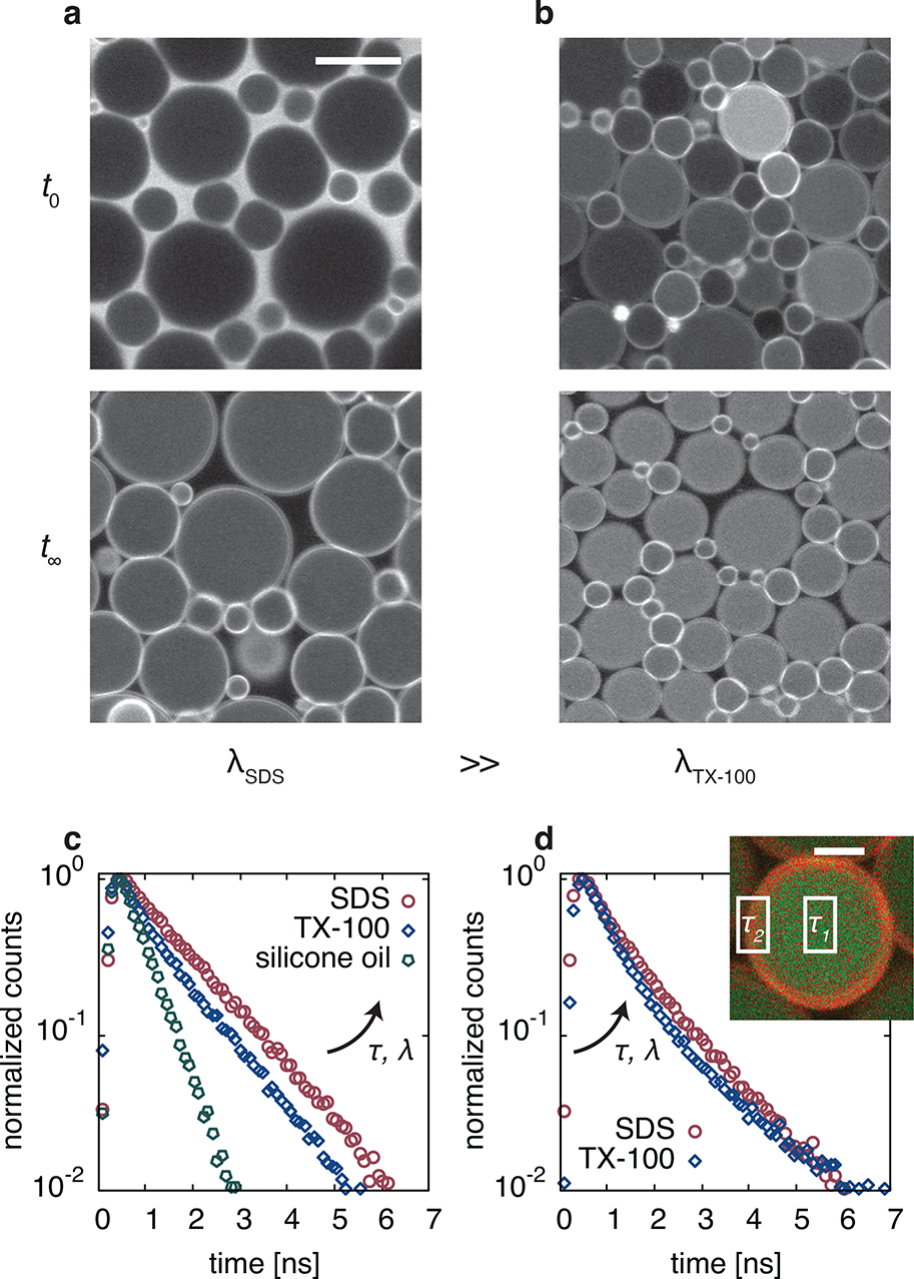
Effect of the surfactant on the molecular transport. (a, b) Fluorescence intensity images of BODIPY-C12 in SDS (a) and TX-100 (b) stabilized emulsions recorded after the emulsions were prepared, at *t*_0_, and after a couple of days when all oil droplets equilibrated to equal dye concentrations, at *t*_∞_. The scale bar is 10 μm. The fluorescence decays of BODIPY-C12 recorded in micellar solutions (c) and at the bright oil–water interface (d) show faster decays for TX-100. The extracted lifetime values are summarized in Table 2. (d, inset) Using the two components found at the oil–water interface, we construct a FLIM image, shown here for a single oil droplet stabilized with TX-100. The fast component corresponds to the oil phase (green), while the slow component shows BODIPY-C12 in the surfactant phase (red). The scale bar is 2 μm.

The degree to which surfactant molecules are hydrophilic or lipophilic can be estimated using their hydrophilic–lipophilic balance (HLB), which is based on the molecular structure of the emulsifier. Using the advanced technique proposed by Guo and co-workers,^38^ we calculate a HLB number of 39.7 for SDS, reflecting its strong hydrophilicity. Substituting SDS by the more hydrophobic TX-100 (compare Figures 1b and 1c) leads to an exchange at the time scale of the emulsion preparation itself because at *t*_0_ the continuous phase is already depleted of BODIPY-C12 (compare Figures 3a and 3b). The TX-100 emulsion was also prepared in clear excess of its critical micelle concentration (at 1 wt %, ≈70 × cmc).^47^ However, substitution of SDS with the structurally similar but more hydrophobic SDBS (compare Figures 1b and 1d) results in hardly any increase in the transport rate (Figure S9). This is surprising considering the comparable HLB values of SDBS and TX-100, which we calculate as 10.7 and 13.7, respectively. To rule out any electrostatic effects slowing down the transport (both SDS and SDBS are anionic), we repeated the experiment with 1 wt % NaCl added to the continuous phase. In this case the time scale of the molecular transport of BODIPY-C12 increases, which indicates a change in the partition coefficient (Figure S9).^16^ These additional experiments suggest that neither the HLB value nor electrostatic effects can explain the increased transport rate of the dye molecule in the case of emulsions stabilized with TX-100.

To get at the core of this, we resort to the analysis of the time-resolved fluorescence of BODIPY-C12 to study its local environments upon exposure to both surfactants, in micellar solutions and at the oil–water interface. The fluorescence decay of BODIPY-C12 was shown to be monoexponential with a lifetime sensitive to viscosity but insensitive to polarity.^18−20,48^ The relationship between fluorescence lifetime τ and macroscopic solvent viscosity η is given by Förster–Hoffmann’s equation τ ∝ *k*η^*x*^, where *k* and *x* are empirical constants obtained from calibration with solvents of known viscosity (Figure S10).^49^ We anticipate the local viscosity to vary with surfactant structure given the presence of large gradients in lateral pressure between the headgroups and tails.^50^ The fluorescence decay curves of BODIPY-C12 in oil, micellar solutions, and at the oil–water interface for both SDS and TX-100 are shown in Figures 3c and 3d, respectively. The extracted (amplitude-weighted average) lifetimes and corresponding viscosities η_loc_, which we determined using Förster–Hoffmann’s equation, are summarized in Table 2. The fluorescence of BODIPY-C12 in micellar solutions of SDS decays monoexponentially with a lifetime τ = 1.28 ns (Figure 3c), indicating that the dye molecules are exposed to a single environment.

**Table 2:** Time-Resolved Fluorescence Parameters^a^

	τ_1_ [ns]	τ_2_ [ns]	*A*_1_/*A*_2_	⟨τ⟩ [ns]	η_loc_ [mPa s]	χ^2^
SDS_aq_	1.28			1.28	55	1.154
TX-100_aq_	0.54	1.37	1.2	0.92	25	0.976
oil	0.52			0.52	6	1.367
SDS_o–w_	0.66	1.56	2.1	0.96	28	1.044
TX-100_o–w_	0.55	1.95	6.1	0.75	15	1.029

a The local viscosities η_loc_ are inferred from the amplitude-weighted average lifetimes, ⟨τ⟩, using a calibration measurement of ethanol–glycerol mixtures (Figure S10).

In this environment, according to our calibration, BODIPY-C12 molecules experience a viscosity of 55 mPa s, which implies a significantly lower mobility of the dye than the one measured in bulk solution at low viscosities (Figure S10). The fluorescence decay of BODIPY-C12 in TX-100 micelles, however, is biexponential, which indicates that the dye molecules probe a second local environment. The amplitude-weighted average lifetime of BODIPY-C12 within TX-100 micelles relates to a lower viscosity of 25 mPa s. Strikingly, the viscosity experienced by BODIPY-C12 in the oil phase appears to be decoupled from the macroscopic viscosity; i.e., the local viscosity η_loc_ is orders of magnitude lower than the one reported from conventional rheometry.^51−53^ This might also explain the lower values for ⟨τ⟩ and η_loc_ at the interface, where the presence of oil inevitably affects the lifetime. Indeed, fitting both components to construct a FLIM image (Figure 3d, inset) reveals that the fast component belongs to the oil phase. Regardless of whether BODIPY-C12 resides in the micellar phase or at the oil–water interface, samples prepared with TX-100 instead of SDS show a local viscosity a factor of 2 lower.

**Figure 4:**
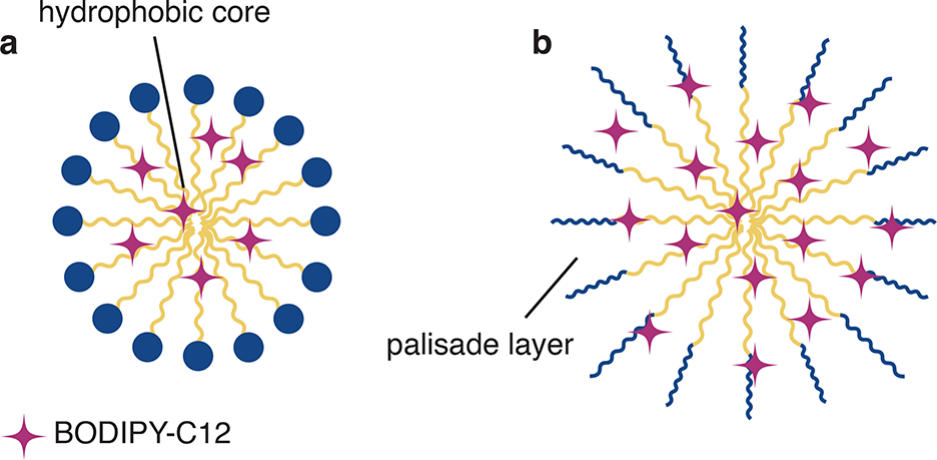
Proposed local environments of BODIPY-C12 in micelles. As suggested by the analysis of the fluorescence decay of micellar solutions, BODIPY-C12 is more localized in SDS micelles (a). In this case, its exchange between micelles and micelles and oil droplets is limited. In the larger TX-100 micelles, however, BODIPY-C12 is less localized and also populates the outer palisade layer (b), which facilitates a fast exchange.

We tentatively interpret these results as follows: given the structural similarities between the surfactant molecules and BODIPY-C12, we hypothesize that the dye molecules intercalate into the micelles. In the case of SDS the dye molecules are subject to a higher degree of molecular crowding and are thus more stable within the macromolecular assembly. Within TX-100 micelles, however, BODIPY-C12 molecules are less localized and more mobile, as suggested by the biexponential decay and significantly lower lifetime. One possible explanation would be that the dye molecules in the TX-100 case are populating both the hydrophobic core of the micelles and their outer palisade layer,^54,55^ which is composed of a poly(ethylene oxide) chain. Hence, for SDS, the exchange to neighboring micelles^56,57^ and oil droplets is more restricted compared to the TX-100 case. These considerations are illustrated in Figure 4. From a structural perspective, the differences in mobility could also be related to differences in the surface density between SDS and TX-100 micelles, where the much less dense surface of TX-100 micelles could facilitate the fast exchange. Additional structural information on the micelle–dye aggregates could for instance be inferred from X-ray studies. We thus conclude that neither the HLB nor electrostatic effects determine the transport rate of the lipophilic dye within this emulsion, but rather the mobility of the dye molecules within the micelle–dye assemblies.

We further substantiate these claims by performing molecular dynamics simulations.

### Simulation Results

In our simulations, we observe the rapid formation of micellar aggregates (composed of dye, oil, and surfactant molecules) in the water phase during the initial 10^5^ time steps. We characterize the formation of these aggregates using the density-based clustering algorithm DBSCAN.^59^ For this clustering we consider O (oil), T (hydrophobic tail), H_s_ (hydrophilic headgroup of the surfactants), or H_d_ (hydrophilic headgroup of the dye) beads (Figure 1) located in the water phase and whose *z*-coordinates are at least 3σ away from the position of the interface. This ensures that the micelles do not interact with the interface when their composition is analyzed. Dye molecules, not participating in the formation of micelles, diffuse freely through the interface. In the experiments, at *t*_0_, the dye molecules are already partitioned into micelles in the aqueous phase. Hence, we excluded the first 10^5^ time steps from the analysis of the diffusion of the dye.

**Figure 5:**
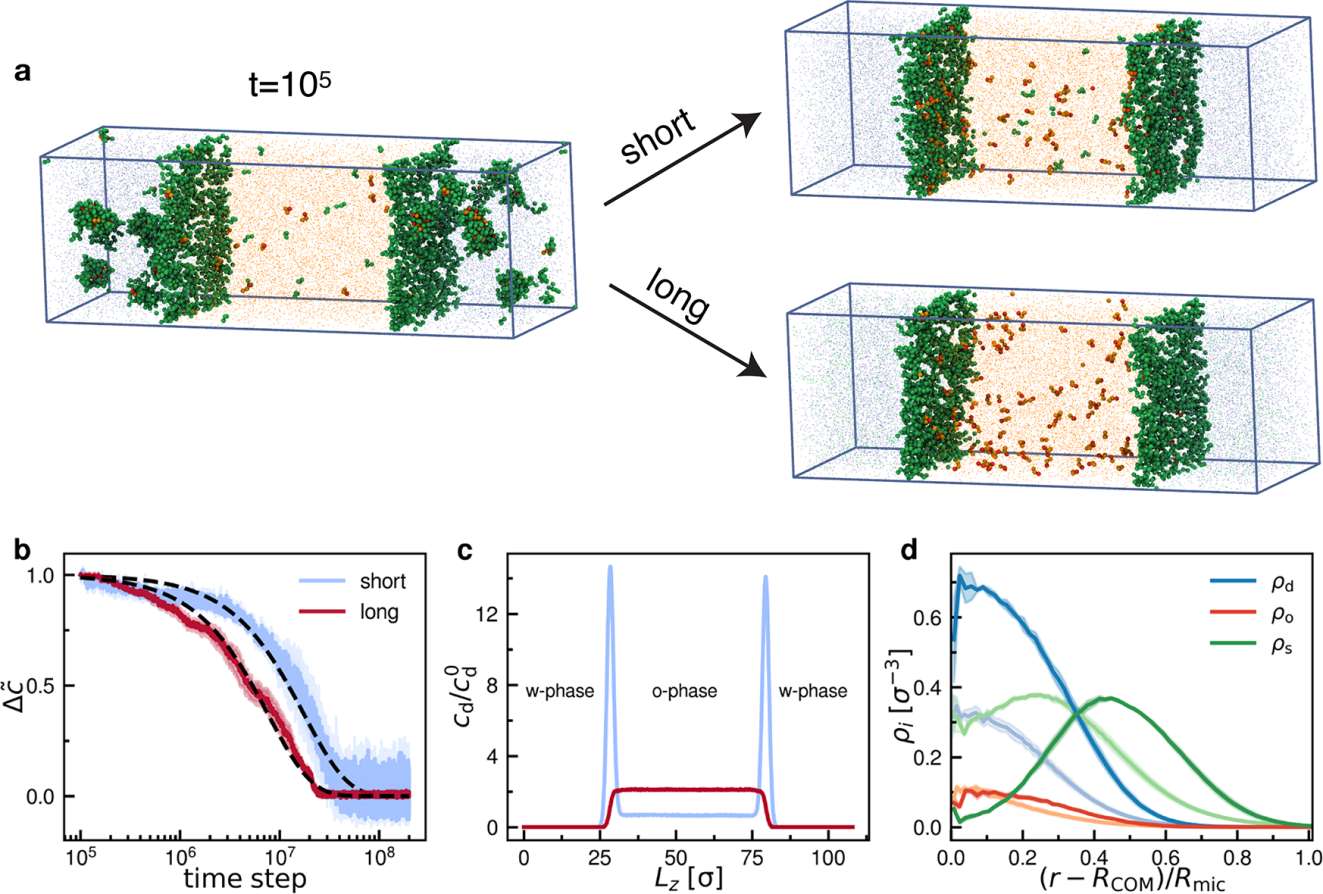
Simulations of the dye diffusion through a oil–water interface decorated by short (*n*_s_ = 3) and long (*n*_s_ = 7) surfactants. (a) Representative simulation snapshots that correspond to the formation of micelles at *t* = 10^5^ time steps and to the end of a simulation. The snapshots have been rendered using Visual Molecular Dynamics.^58^ Dye molecules are colored red (H_d_) and orange (T), surfactant chains are colored green, and oil and water beads are colored orange and blue, respectively. (b) Temporal evolution of the normalized concentration difference Δ*c̃* of dye molecules present in both water and oil phases. The data are fit using e^–*t*/λ^ (dashed lines). (c) Concentration profiles *c*_d_/*c*_d_^0^ of beads belonging to dye molecules along the *z*-axis normalized by the bulk values. (d) Number density of beads belonging to a dye (ρ_d_), oil (ρ_o_), or surfactant molecule (ρ_s_) with respect to the radial distance from the center of mass of a micelle, *R*_COM_, normalized by its size, *R*_mic_. The light and dark curves correspond to systems with short and long surfactant chains, respectively. Shaded areas represent the error bars, calculated as the standard error of the mean from three independent realizations.

Figure 5a shows representative snapshots of the systems investigated, at the time of the micelle formation and at the end of a simulation. Figure 5b shows a plot of the temporal evolution of the normalized concentration difference Δ*c̃* of the dye molecules present in the water and oil phase using the same definition for Δ*c̃*(*t*) as employed in the experiments.

The simulation runs between Δ*c*_0_(*t* = 10^5^) and Δ*c*_*∞*_(*t* = 2 × 10^8^) and recovers the exponential decay (eq 2) that we also observed in the experiments (Figure 2d,e). The results suggest a slower exchange for the diffusion of solutes in systems where the interface consists of short surfactant chains than in the ones with long surfactant chains. The characteristic time scale obtained from the fitting reveals that for the short surfactant chains λ^short^ = 1.8 × 10^7^ time steps is more than two times larger than the value for long surfactant chains λ^long^ = 7.7 × 10^6^ time steps. These findings are in good qualitative agreement with the experimental results (Figure 3a,b). Thus, our computational results suggest that the energetic penalty to cross the interface decorated by the long surfactant is lower than for the interface decorated by short surfactant as supported by the temporal evolution of the nonbonded potential energy in the system (Figure S11). For a more quantitative description, calculations of the transfer free energy of a dye molecule from an aqueous to an oil phase through the interface decorated by two surfactant types could be performed.

Next, we compute the concentration profile of beads belonging to dye molecules along the *z*-axis (Figure 5c). For systems composed of surfactants with long headgroups, dye molecules are homogeneously distributed inside the oil phase. In contrast, dye molecules are more prone to stick to the interface when the headgroup of the surfactant is small. We note that due to a rather small number (*N*_d_ = 100) of dye molecules simulated and on the time scales accessible in the simulations, the difference in the concentration profiles between these systems is amplified. Qualitatively, it resembles the experiments at the early stage, where highly fluorescent regions at the periphery of the droplets were observed (Figure 2b). Experimentally, such aggregation was shown to occur for a variety of molecules, independent of charge, and could be attributed to the presence of a gradient in the electric field at the oil–water interface.^44^ For future studies, it could be interesting to elucidate the mechanism behind the aggregation in more detail, for instance, by performing free energy calculations as described above or by performing neutron or X-ray scattering experiments.

Finally, we study the composition of the micelles by calculating the number density profiles of each component. For both simulated systems, the micelles are predominantly made up of dye and surfactant beads, with a small number of oil beads present at the core (Figure 5d). We find a comparable amount of surfactant beads in both simulated systems and that the density of dye beads is higher for systems containing weakly interacting headgroups. Consequently, clusters containing surfactant molecules with long headgroups are less stable against dissolution; this results in a faster diffusion of dye molecules into the oil phase, which is in line with our experimental results, showing a faster molecular transport of the dye molecule for emulsions stabilized with TX-100 (long headgroups) than for the ones stabilized with SDS (short headgroups).

## Conclusion

To conclude, we observe experimentally that the molecular transport of a lipophilic dye molecule in a model oil-in-water emulsion is a two-step process that depends on droplet size, surfactant concentration, and surfactant type, for which we suggest simple models. For some surfactants, this transport can be limited by diffusion through the interface, a phenomenon that we find to be independent of both the hydrophilic–lipophilic balance of the surfactant molecules and the presence of electrostatic effects. Instead, the analysis of the time-resolved fluorescence of the molecular rotor suggests that the mobility of the dye molecules within the micelles plays a role; micelle–dye assemblies consisting of surfactant molecules with smaller headgroups stabilize the dye molecules against dissolution into the oil phase. Our findings are supported by molecular dynamics simulations, which recover the behavior of dye depletion from the continuous phase. They show that there is indeed a strong dependence of this molecular transport on the molecular size of the surfactant molecules stabilizing the oil–water interface. We believe these results can be valuable for designing any application in which emulsions are being used as compartments, particularly for drug delivery.^60^

## Supporting Information Available

Emulsion stability, BODIPY-C12 solubility, BODIPY-C12 intensity–concentration dependence, concentration profile of surfactant beads along the *z*-axis, DLS measurements of micelles with/and without the addition of BODIPY-C12, depletion of the continuous phase, oil droplet size dependence of the molecular transport, influence of HLB and salt, calibration of BODIPY-C12, image analysis (PDF)
Video S1 (MPG)
Video S2 (MPG)

## Supplementary Material

la2c02597_si_001.pdf

la2c02597_si_002.mpg

la2c02597_si_003.mpg

## References

[ref1] Leal-Calderon F.; Schmitt V.; Bibette J. Emulsion Science: Basic Principles; Springer Science & Business Media: 2007.

[ref2] Buyukozturk F.; Benneyan J. C.; Carrier R. L. Impact of emulsion-based drug delivery systems on intestinal permeability and drug release kinetics. Journal of controlled release 2010, 142, 22–30. 10.1016/j.jconrel.2009.10.005. 19850092

[ref3] McClements D. J. Nanoemulsions versus microemulsions: terminology, differences, and similarities. Soft Matter 2012, 8, 1719–1729. 10.1039/C2SM06903B.

[ref4] Wadhwa J.; Nair A.; Kumria R. Emulsion forming drug delivery system for lipophilic drugs. Acta Polym. Pharm. 2012, 69, 179–91. 22568032

[ref5] McClements D. J.; Decker E. A.; Weiss J. Emulsion-based delivery systems for lipophilic bioactive components. J. Food Sci. 2007, 72, R109–R124. 10.1111/j.1750-3841.2007.00507.x. 17995616

[ref6] Porter C. J.; Trevaskis N. L.; Charman W. N. Lipids and lipid-based formulations: optimizing the oral delivery of lipophilic drugs. Nat. Rev. Drug Discovery 2007, 6, 231–248. 10.1038/nrd2197. 17330072

[ref7] Waring M. J. Lipophilicity in drug discovery. Expert Opinion on Drug Discovery 2010, 5, 235–248. 10.1517/17460441003605098. 22823020

[ref8] Arnott J. A.; Planey S. L. The influence of lipophilicity in drug discovery and design. Expert opinion on drug discovery 2012, 7, 863–875. 10.1517/17460441.2012.714363. 22992175

[ref9] Hiranphinyophat S.; Otaka A.; Asaumi Y.; Fujii S.; Iwasaki Y. Particle-stabilized oil-in-water emulsions as a platform for topical lipophilic drug delivery. Colloids Surf., B 2021, 197, 111423 10.1016/j.colsurfb.2020.111423. 33142258

[ref10] Kabalnov A.; Pertzov A.; Shchukin E. Ostwald ripening in emulsions: I. Direct observations of Ostwald ripening in emulsions. J. Colloid Interface Sci. 1987, 118, 590–597. 10.1016/0021-9797(87)90492-9.

[ref11] Taylor P. Ostwald ripening in emulsions. Advances in colloid and interface science 1998, 75, 107–163. 10.1016/S0001-8686(98)00035-9. 14672850

[ref12] Fletcher P. D.; Howe A. M.; Robinson B. H. The kinetics of solubilisate exchange between water droplets of a water-in-oil microemulsion. J. Chem. Soc., Faraday Transactions 1: Physical Chemistry in Condensed Phases 1987, 83, 985–1006. 10.1039/f19878300985.

[ref13] Courtois F.; Olguin L. F.; Whyte G.; Theberge A. B.; Huck W. T.; Hollfelder F.; Abell C. Controlling the retention of small molecules in emulsion microdroplets for use in cell-based assays. Analytical chemistry 2009, 81, 3008–3016. 10.1021/ac802658n. 19284775

[ref14] Chen Y.; Gani A. W.; Tang S. K. Characterization of sensitivity and specificity in leaky droplet-based assays. Lab Chip 2012, 12, 5093–5103. 10.1039/c2lc40624a. 23090153

[ref15] Skhiri Y.; Gruner P.; Semin B.; Brosseau Q.; Pekin D.; Mazutis L.; Goust V.; Kleinschmidt F.; El Harrak A.; Hutchison J. B.; et al. Dynamics of molecular transport by surfactants in emulsions. Soft Matter 2012, 8, 10618–10627. 10.1039/c2sm25934f.

[ref16] Gruner P.; Riechers B.; Semin B.; Lim J.; Johnston A.; Short K.; Baret J.-C. Controlling molecular transport in minimal emulsions. Nat. Commun. 2016, 7, 1–9. 10.1038/ncomms10392. PMC4735829 26797564

[ref17] Etienne G.; Vian A.; Biočanin M.; Deplancke B.; Amstad E. Cross-talk between emulsion drops: how are hydrophilic reagents transported across oil phases?. Lab Chip 2018, 18, 3903–3912. 10.1039/C8LC01000E. 30465575

[ref18] Kuimova M. K. Mapping viscosity in cells using molecular rotors. Phys. Chem. Chem. Phys. 2012, 14, 12671–12686. 10.1039/c2cp41674c. 22806312

[ref19] Wu Y.; Štefl M.; Olzyńska A.; Hof M.; Yahioglu G.; Yip P.; Casey D. R.; Ces O.; Humpolíčková J.; Kuimova M. K. Molecular rheometry: direct determination of viscosity in L o and L d lipid phases via fluorescence lifetime imaging. Phys. Chem. Chem. Phys. 2013, 15, 14986–14993. 10.1039/c3cp51953h. 23912893

[ref20] López-Duarte I.; Vu T. T.; Izquierdo M. A.; Bull J. A.; Kuimova M. K. A molecular rotor for measuring viscosity in plasma membranes of live cells. Chem. Commun. 2014, 50, 5282–5284. 10.1039/C3CC47530A. 24266030

[ref21] Mukerjee P.; Mysels K. J. Critical Micelle Concentrations of Aqueous Surfactant Systems, 1971.

[ref22] Paredes J.; Michels M. A.; Bonn D. Rheology across the zero-temperature jamming transition. Physical review letters 2013, 111, 015701 10.1103/PhysRevLett.111.015701. 23863014

[ref23] Dekker R. I.; Dinkgreve M.; de Cagny H.; Koeze D. J.; Tighe B. P.; Bonn D. Scaling of flow curves: Comparison between experiments and simulations. J. Non-Newtonian Fluid Mech. 2018, 261, 33–37. 10.1016/j.jnnfm.2018.08.006.

[ref24] Wagner R. W.; Lindsey J. S. Boron-dipyrromethene dyes for incorporation in synthetic multi-pigment light-harvesting arrays. Pure Appl. Chem. 1996, 68, 1373–1380. 10.1351/pac199668071373. PMC12826650 41585346

[ref25] Hosny N. A.; Mohamedi G.; Rademeyer P.; Owen J.; Wu Y.; Tang M.-X.; Eckersley R. J.; Stride E.; Kuimova M. K. Mapping microbubble viscosity using fluorescence lifetime imaging of molecular rotors. Proc. Natl. Acad. Sci. U. S. A. 2013, 110, 9225–9230. 10.1073/pnas.1301479110. 23690599 PMC3677502

[ref26] Kang J.; Lhee S.; Lee J. K.; Zare R. N.; Nam H. G. Restricted intramolecular rotation of fluorescent molecular rotors at the periphery of aqueous microdroplets in oil. Sci. Rep. 2020, 10, 1–10. 10.1038/s41598-020-73980-7. 33033365 PMC7545199

[ref27] Schindelin J.; Arganda-Carreras I.; Frise E.; Kaynig V.; Longair M.; Pietzsch T.; Preibisch S.; Rueden C.; Saalfeld S.; Schmid B.; et al. Fiji: an open-source platform for biological-image analysis. Nat. Methods 2012, 9, 676–682. 10.1038/nmeth.2019. 22743772 PMC3855844

[ref28] Legland D.; Arganda-Carreras I.; Andrey P. MorphoLibJ: integrated library and plugins for mathematical morphology with ImageJ. Bioinformatics 2016, 32, 3532–3534. 10.1093/bioinformatics/btw413. 27412086

[ref29] Wagner T.; Eglinger J. thorstenwagner/ij-ellipsesplit: EllipseSplit 0.6.0 SNAPSHOT, 2017.

[ref30] Bishop M.; Kalos M.; Frisch H. Molecular dynamics of polymeric systems. J. Chem. Phys. 1979, 70, 1299–1304. 10.1063/1.437567.

[ref31] Roy S.; Dietrich S.; Höfling F. Structure and dynamics of binary liquid mixtures near their continuous demixing transitions. J. Chem. Phys. 2016, 145, 134505 10.1063/1.4963771. 27782419

[ref32] Diaz-Herrera E.; Ramirez-Santiago G.; Moreno-Razo J. A. Phase and interfacial behavior of partially miscible symmetric Lennard-Jones binary mixtures. J. Chem. Phys. 2005, 123, 184507 10.1063/1.2102787. 16292914

[ref33] Morozova T. I.; Nikoubashman A. Surface Activity of Soft Polymer Colloids. Langmuir 2019, 35, 16907–16914. 10.1021/acs.langmuir.9b03202. 31789037

[ref34] Grest G. S.; Kremer K. Molecular dynamics simulation for polymers in the presence of a heat bath. Phys. Rev. A 1986, 33, 3628 10.1103/PhysRevA.33.3628. 9897103

[ref35] Vu T. V.; Papavassiliou D. V. Oil-water interfaces with surfactants: A systematic approach to determine coarse-grained model parameters. J. Chem. Phys. 2018, 148, 204704 10.1063/1.5022798. 29865808

[ref36] Liese S.; Gensler M.; Krysiak S.; Schwarzl R.; Achazi A.; Paulus B.; Hugel T.; Rabe J. P.; Netz R. R. Hydration effects turn a highly stretched polymer from an entropic into an energetic spring. ACS Nano 2017, 11, 702–712. 10.1021/acsnano.6b07071. 27977927

[ref37] Zhang X.-F.; Zhu J. BODIPY parent compound: fluorescence, singlet oxygen formation and properties revealed by DFT calculations. J. Lumin. 2019, 205, 148–157. 10.1016/j.jlumin.2018.09.017.

[ref38] Guo X.; Rong Z.; Ying X. Calculation of hydrophile–lipophile balance for polyethoxylated surfactants by group contribution method. J. Colloid Interface Sci. 2006, 298, 441–450. 10.1016/j.jcis.2005.12.009. 16414065

[ref39] Ren Y.; Zhang Q.; Yang N.; Xu J.; Liu J.; Yang R.; Kunkelmann C.; Schreiner E.; Holtze C.; Mülheims K.; et al. Molecular dynamics simulations of surfactant adsorption at oil/water interface under shear flow. Particuology 2019, 44, 36–43. 10.1016/j.partic.2018.09.002.

[ref40] Kanellopoulos A.; Owen M. Adsorption of sodium dodecyl sulphate at the silicone fluid/water interface. Trans. Faraday Soc. 1971, 67, 3127–3138. 10.1039/tf9716703127.

[ref41] Anderson J. A.; Glaser J.; Glotzer S. C. HOOMD-blue: A Python package for high-performance molecular dynamics and hard particle Monte Carlo simulations. Comput. Mater. Sci. 2020, 173, 109363 10.1016/j.commatsci.2019.109363.

[ref42] Osakai T.; Yamada H.; Nagatani H.; Sagara T. Potential-dependent adsorption of amphoteric rhodamine dyes at the oil/water interface as studied by potential-modulated fluorescence spectroscopy. J. Phys. Chem. C 2007, 111, 9480–9487. 10.1021/jp0723315.

[ref43] Zhou Z.; Yan X.; Lai Y.-H.; Zare R. N. Fluorescence polarization anisotropy in microdroplets. journal of physical chemistry letters 2018, 9, 2928–2932. 10.1021/acs.jpclett.8b01129. 29763551

[ref44] Xiong H.; Lee J. K.; Zare R. N.; Min W. Strong concentration enhancement of molecules at the interface of aqueous microdroplets. J. Phys. Chem. B 2020, 124, 9938–9944. 10.1021/acs.jpcb.0c07718. 33084345

[ref45] Zwolinski B. J.; Eyring H.; Reese C. E. Diffusion and Membrane Permeability. J. Phys. Chem. 1949, 53, 1426–1453. 10.1021/j150474a012.

[ref46] Bruce C. D.; Berkowitz M. L.; Perera L.; Forbes M. D. Molecular dynamics simulation of sodium dodecyl sulfate micelle in water: micellar structural characteristics and counterion distribution. J. Phys. Chem. B 2002, 106, 3788–3793. 10.1021/jp013616z.

[ref47] Tiller G. E.; Mueller T. J.; Dockter M. E.; Struve W. G. Hydrogenation of Triton X-100 eliminates its fluorescence and ultraviolet light absorption while preserving its detergent properties. Analytical biochemistry 1984, 141, 262–266. 10.1016/0003-2697(84)90455-X. 6496933

[ref48] Chung P.-H.; Levitt J. A.; Kuimova M. K.; Yahioglu G.; Suhling K. Mapping intracellular viscosity by advanced fluorescence imaging of molecular rotors in living cells. Multiphoton Microscopy in the Biomedical Sciences XI, 2011; p 790323.

[ref49] Förster T.; Hoffmann G. Die Viskositätsabhängigkeit der Fluoreszenzquantenausbeuten einiger Farbstoffsysteme. Zeitschrift für Physikalische Chemie 1971, 75, 63–76. 10.1524/zpch.1971.75.1_2.063.

[ref50] Seddon A. M.; Casey D.; Law R. V.; Gee A.; Templer R. H.; Ces O. Drug interactions with lipid membranes. Chem. Soc. Rev. 2009, 38, 2509–2519. 10.1039/b813853m. 19690732

[ref51] Vu T. T.; Méallet-Renault R.; Clavier G.; Trofimov B. A.; Kuimova M. K. Tuning BODIPY molecular rotors into the red: sensitivity to viscosity vs. temperature. J. Mater. Chem. C 2016, 4, 2828–2833. 10.1039/C5TC02954F.

[ref52] Polita A.; Toliautas S.; Žvirblis R.; Vyšniauskas A. The effect of solvent polarity and macromolecular crowding on the viscosity sensitivity of a molecular rotor BODIPY-C 10. Phys. Chem. Chem. Phys. 2020, 22, 8296–8303. 10.1039/C9CP06865A. 32103227

[ref53] Bittermann M. R.; Grzelka M.; Woutersen S.; Brouwer A. M.; Bonn D. Disentangling nano-and macroscopic viscosities of aqueous polymer solutions using a fluorescent molecular rotor. J. Phys. Chem. Lett. 2021, 12, 3182–3186. 10.1021/acs.jpclett.1c00512. 33759527 PMC8041377

[ref54] Kumbhakar M.; Nath S.; Mukherjee T.; Pal H. Solvation dynamics in triton-X-100 and triton-X-165 micelles: effect of micellar size and hydration. J. Chem. Phys. 2004, 121, 6026–6033. 10.1063/1.1784774. 15367031

[ref55] Kumbhakar M.; Goel T.; Mukherjee T.; Pal H. Nature of the water molecules in the palisade layer of a triton X-100 micelle in the presence of added salts: A solvation dynamics study. J. Phys. Chem. B 2005, 109, 14168–14174. 10.1021/jp0520291. 16852779

[ref56] Rharbi Y.; Winnik M. A. Solute exchange between surfactant micelles by micelle fragmentation and fusion. Adv. Colloid Interface Sci. 2001, 89, 25–46. 10.1016/S0001-8686(00)00054-3. 11215796

[ref57] Rharbi Y.; Winnik M. A. Salt effects on solute exchange in sodium dodecyl sulfate micelles. J. Am. Chem. Soc. 2002, 124, 2082–2083. 10.1021/ja0123397. 11878939

[ref58] Humphrey W.; Dalke A.; Schulten K. VMD: visual molecular dynamics. J. Mol. Graphics 1996, 14, 33–38. 10.1016/0263-7855(96)00018-5. 8744570

[ref59] Pedregosa F.; et al. Scikit-learn: Machine Learning in Python. Journal of Machine Learning Research 2011, 12, 2825–2830.

[ref60] Lu G. W.; Gao P. Handbook of Non-invasive Drug Delivery Systems; Elsevier: 2010; pp 59–94.

