# Supplementary material for: Surface-Mediated Molecular Transport of a Lipophilic Fluorescent Probe in Polydisperse Oil-in-Water Emulsions
Source: Langmuir. 2023 Mar 15;39(12):4207–15. doi: 10.1021/acs.langmuir.2c02597 (PMC10061922; doi:10.1021/acs.langmuir.2c02597)
Supplement: Supplementary file 1 — la2c02597_si_001.pdf [file la2c02597_si_001.pdf]

Supporting Information for Surface-Mediated Molecular Transport of a Lipophilic Fluorescent Probe in Polydisperse Oil-in-Water Emulsions

Marius R. Bittermann,[1,*] Tatiana I. Morozova,[2,†] Santiago F. Velandia,[1]
Elham Mirzahossein,[1] Antoine Deblais,[1] Sander Woutersen,[3] and Daniel Bonn[1,‡]
[1] *Van der Waals-Zeeman Institute, IoP, University of Amsterdam, Science Park 904, 1098 XH Amsterdam, Netherlands.*
[2] *Institut Laue-Langevin, 71 Avenue des Martyrs, Grenoble 38042, France*
[3] *Van 't Hoff Institute for Molecular Sciences, University of Amsterdam, Science Park 904, 1098 XH Amsterdam, Netherlands.*

Emulsion Stability

The droplet sizes of the emulsions are shown in Fig. S1 and remained constant over time.

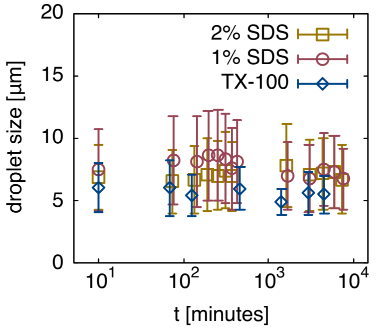

FIG. S1. Droplet size as a function of time for different surfactant types and concentrations.

BODIPY-C12 Solubility

Fluorescence microscopy intensity images (Fig. S2) show that for micellar solutions below the cmc, BODIPY-C12 forms aggregates and is poorly soluble. Above the cmc, however, the intensity is homogeneous. This suggests that BODIPY-C12 is solubilized by micelles.

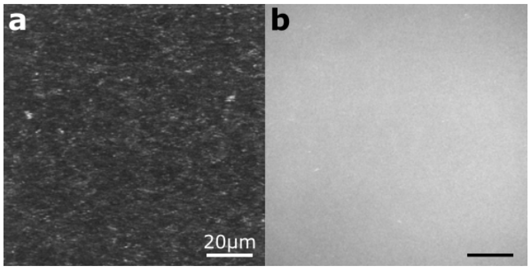

FIG. S2. Fluorescence intensity images of BODIPY in SDS solutions below the cmc (0.1%, a) and above the cmc (1%, b).

BODIPY-C12 Intensity-Concentration Dependence

To verify the dependency of the intensity of BODIPY-C12 on its concentration we carried out a reference measurement in 1% SDS solution, in which we varied the concentration of the fluorophore. Fig. S3 shows an approximately linear relationship.

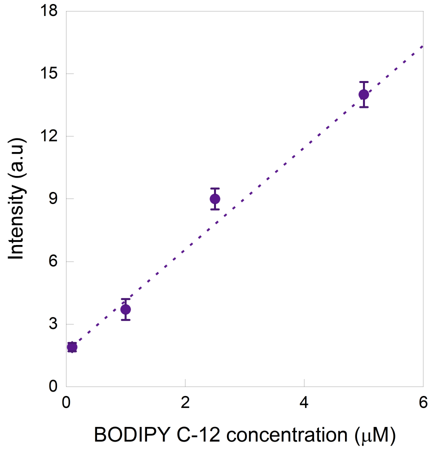

FIG. S3. Fluorescence intensity plotted versus fluorophore concentration shown for a solution containing 1% SDS. The fit is linear.

* m.r.bittermann@uva.nl
† morozova@ill.fr
‡ d.bonn@uva.nl

Concentration Profile of Surfactant Beads Along the z-Axis

In Fig. S4 we plot the concentration profile of surfactant beads along the z-axis for two surfactant architectures, i.e. chains composed of three beads (short) and seven (long) beads.

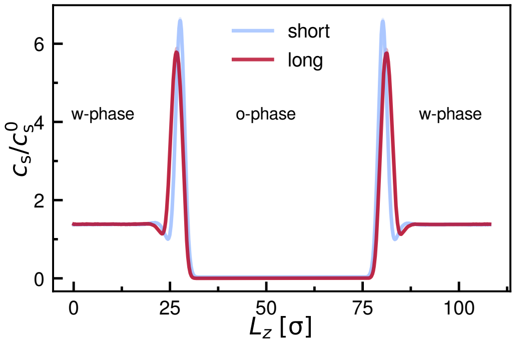

FIG. S4. Concentration profile $c_{\rm s}/c_{\rm s}^0$ of beads belonging to surfactant along the z-axis normalized by its bulk value for short ($n_{\rm s} = 3$) and long ($n_{\rm s} = 7$) chains investigated. The interaction strength $\varepsilon_{\rm H_sH_d}/\varepsilon$ equals to 3.6 and 0.225 for a short and a long surfactant chain, respectively.

DLS Measurements of Micelles with/and without the Addition of BODIPY-C12

The effect of the addition of BODIPY-C12 to the micellar size of SDS and TX-100 is demonstrated by DLS measurements (Fig. S5). In both cases, the fluorophore causes the micelles to become bigger, which indicates that the micelles are swollen with BODIPY-C12.

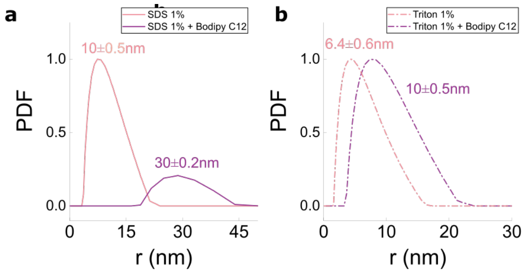

FIG. S5. DLS measurements of micellar solutions of SDS and TX-100. The addition of BODIPY-C12 shifts the size distributions to bigger radii.

Depletion of the Continuous Phase

Fig. S6 shows the decrease of fluorescence intensity of BODIPY-C12 in the continuous phase of SDS stabilized emulsions. Given that SDS micelles are insoluble in oil, we surmise the decrease in intensity to originate from a decrease in dye concentration. From day 2 onwards, the fluorophore concentration in the micellar phase remains constant.

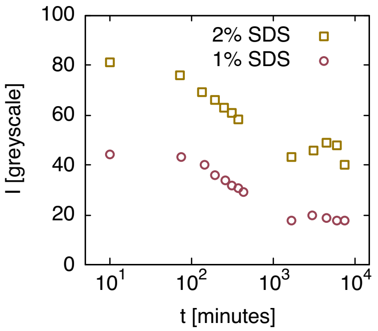

FIG. S6. Raw data of the fluorescence intensity of BODIPY C-12 in the continuous phase. The concentration of BODIPY-C12 in the continuous phase decreases only on the first day of the measurement.

Molecular Transport in a Non-emulsified System

To investigate whether the molecular transport of BODIPY-C12 is limited to emulsified systems, we carried out additional experiments, in which we measured the transport in non-emulsified systems. For this we squeezed 0.2 mL of micellar phase (containing 1% and 2% SDS, and $\approx 1\,\mu$M BODIPY-C12) surrounded by 0.8 mL of oil between two glass slides separated by 300 μm (using spacers) and measured the fluorescence intensity and lifetime analogously to the emulsified system (Fig. S7, shown here for 1% SDS). We find that, after one week (t_∞ of the emulsified system) the concentration of dye in the system containing 1% SDS (Intensity, $I = 5.41 \pm 0.1$) exceeds the concentration of dye in the system containing 2% SDS ($I = 2.52 \pm 0.2$) by a factor ≈ 2, similar to the ratio of the partition coefficients inferred from the emulsified systems. The measured lifetimes in the oil phase were $\tau = 0.546 \pm 0.01$ ns and $\tau = 0.562 \pm 0.02$ ns for the samples prepared with 1% SDS and 2% SDS, respectively. These values are expected and in line with the lifetimes measured in the oil droplets. However, this bulk system is not at equilibrium yet, indicated by (i) the presence of a diffusion front propagating from the water phase, and (ii) the water phase being brighter than the oil phase. We thus surmise that the transport can also be observed in bulk, albeit on a much slower rate.

Oil Droplet Size Dependence of the Molecular Transport

A plot of Δc versus droplet size for different time steps and concentrations of SDS (Fig. S8a, b) reveals that Δc

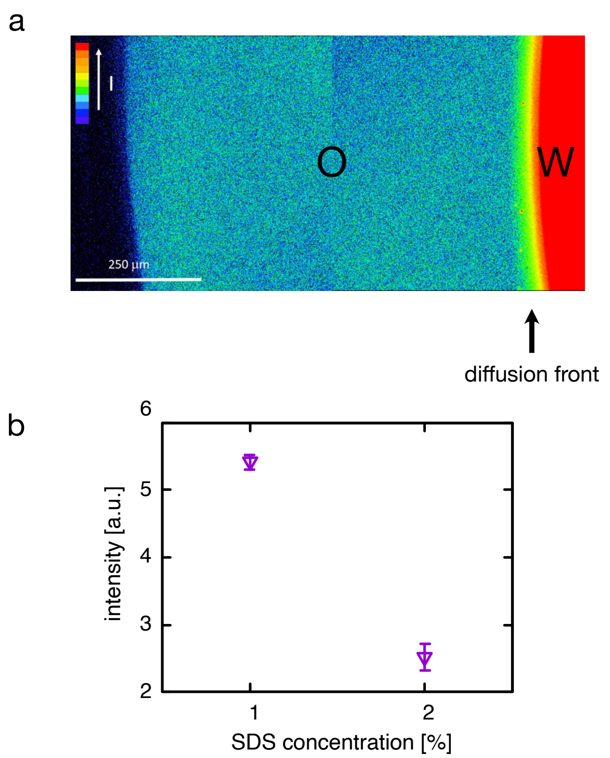

FIG. S7. (a) Diffusion of BODIPY-C12 from micelles (W, 1% SDS) into oil (O) in the non-emulsified equivalent of our experiment. The fluorescence intensity image was recorded after one week. The transport happens much slower, as highlighted by the presence of a diffusion front emanating from the intense water phase. (b) The intensities of the oil phases differ by a factor of ≈ 2.

increases with droplet size, d, but decreases in time. Interestingly, Δc also increases with surfactant concentration. For further data analysis we binned the data into four intervals to fix the droplet size. To mitigate the potential effect of oil droplet size on the intensity signal, as caused by the non-transparency of the emulsions, we kept the range of investigated droplet sizes small, from $4 - 12\,\mu m$.

Influence of HLB and Salt

To study the influence of a lower hydrophilic-lipophilic balance (HLB) and salt on the dynamics of the transport process we repeated the experiments with 1 wt% SDBS ($\approx$ 76 $\times$ cmc) [1], and 1 wt% SDS + 1 wt% NaCl, respectively. Both cases do not significantly speed up the molecular transport, as shown in Fig. S9.

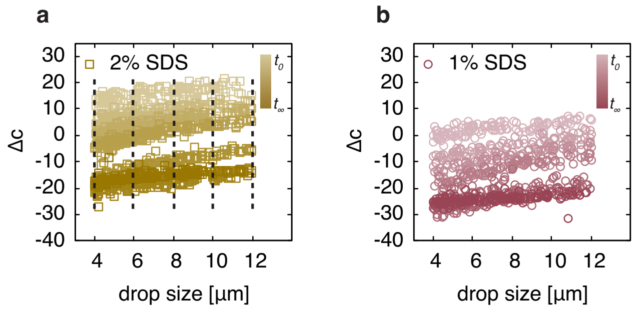

FIG. S8. (a, b) A plot of Δc versus oil droplet size for different time steps (color gradient) and concentration of surfactant. The data is binned into four intervals to fix the drop size (dashed lines).

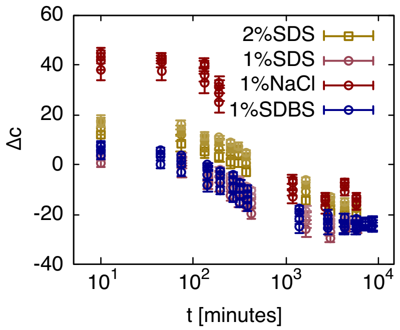

FIG. S9. Dynamics of the molecular transport of BODIPY-C12 from the continuous phase into oil droplets. Lowering the HLB value or adding salt to SDS does not slow down the timescale related to the transport process.

Calibration of BODIPY-C12

According to Förster-Hoffmann's equation the fluorescence lifetime τ of a molecular rotor scales with solvent viscosity η as $\tau \propto k\eta^x$. To calibrate the molecular rotor we prepared ethanol-glycerol solutions with BODIPY-C12 at $\approx 1\,\mu$M and measured the fluorescence lifetime of the solutions. The solvent viscosities were measured using an Anton Paar MCR 302 rheometer with a cone-plate of 50 mm diameter at an angle of $1\,°$. From the calibration curve (Fig. S10) we obtained $k = 0.24 \pm 0.07$ and $x = 0.42 \pm 0.05$.

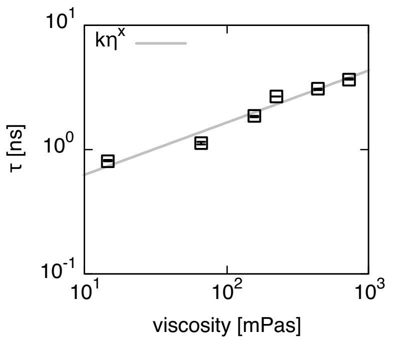

FIG. S10. Calibration curve of BODIPY-C12 in ethanol-glycerol mixtures. The fluorescence lifetime of BODIPY-C12 increases with solvent viscosity.

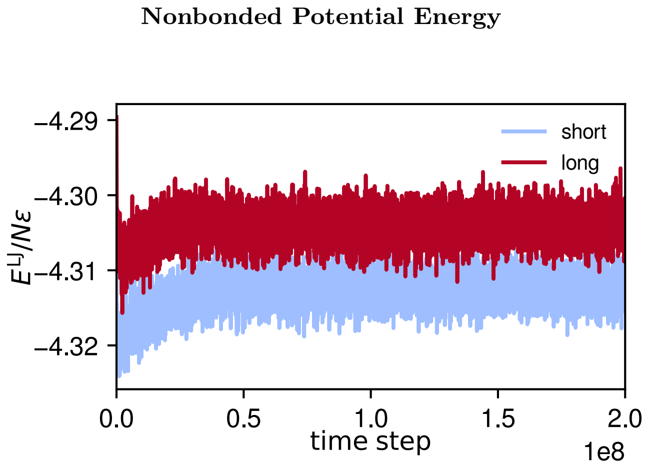

FIG. S11. Nonbonded potential energy per particle, $U^{\mathrm{LJ}}/N\varepsilon$, as a function of the simulation time step for systems containing short and long surfactant chains.

Image Analysis

Image analysis was carried out using Fiji [2] together with MorpholibJ [3] and the ellipse splitting plugin [4]. Within Fiji we wrote a macro based on the following processes; first the background was subtracted followed by the application of a Gaussian Blur filter. Before binarizing the image by applying a (auto-)threshold we enhanced the local contrast using CLAHE. Then we applied dilation as a morphological filter. Eventually, we used the ellipse splitting plugin to detect the droplets (excluding the ones on the side), from which we extracted the Feret's Diameter and the mean intensity. Intensities were measured from the unprocessed images. This workflow worked well for our system, as shown in Fig. S12.

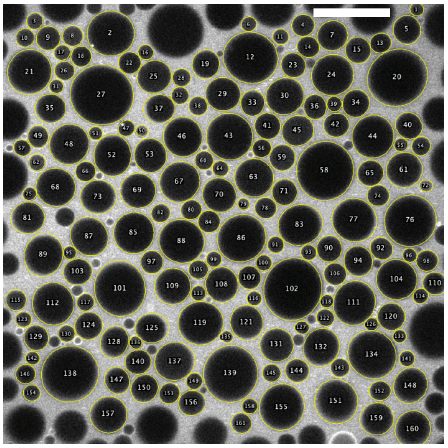

FIG. S12. Image analysis based on Fiji. The image processing workflow as described above detects the droplets well. The example shown here is for the emulsion stabilized with 1% SDS, at t_0. Scale bar is 20 µm.

Supporting Videos

In addition to this supporting information we also provide videos of our simulations as supplementary material, which show the diffusion processes for both (short, long-chains) types of surfactant studied. Surfactants (for both types) are rendered as small green beads, while dye molecules are rendered as orange (tail) and red (head) beads. The movies clearly show that for short surfactant chains, the dye molecules are retained at the interface and their diffusion is slowed down. In in the case of long surfactant chains, however, the beads cross the surfactant phase much faster, and without inhibition.

REFERENCES

[1] S. W. H. Shah, B. Naseem, W. Rehman, N. Bashir, and S. S. Shah, Investigation of 1-alkanols in organised solutions, Bulletin of the Chemical Society of Ethiopia **25** (2011).

[2] J. Schindelin, I. Arganda-Carreras, E. Frise, V. Kaynig, M. Longair, T. Pietzsch, S. Preibisch, C. Rueden, S. Saalfeld, B. Schmid, *et al.*, Fiji: an open-source platform for biological-image analysis, Nature methods **9**, 676 (2012).

[3] D. Legland, I. Arganda-Carreras, and P. Andrey, Morpholibj: integrated library and plugins for mathematical morphology with imagej, Bioinformatics **32**, 3532 (2016).

[4] T. Wagner and J. Eglinger, thorstenwagner/ij-ellipsesplit: EllipseSplit 0.6.0 SNAPSHOT (2017).
